# Preliminary comparative study of lower extremity pressure measurements under the conditions using former models and new lithotomy stirrups in rectal cancer surgery

**DOI:** 10.1186/s12957-024-03352-2

**Published:** 2024-04-03

**Authors:** Takayuki Ochi, Hidetoshi Katsuno, Hiroyuki Kato, Shinya Takagi, Kenji Kikuchi, Kenichi Nakamura, Tomoyoshi Endo, Kazuhiro Matsuo, Hironobu Yasuoka, Akihiro Nishimura, Akihiko Horiguchi, Zenichi Morise

**Affiliations:** 1 Department of Gastroenterological Surgery, Fujita Health University School of Medicine, Bantane Hospital, Nagoya, Japan; 2 Department of Surgery, Fujita Health University School of Medicine Okazaki Medical Center, Okazaki, Japan

**Keywords:** Lithotomy stirrups-2, Well-leg compartment syndrome, Deep venous thrombosis, Robot-assisted rectal surgery

## Abstract

**Background:**

This study aimed to investigate the effect of the use of new lithotomy stirrups-2 on the pressure dispersal on lower limbs, which may lead to the prevention of well-leg compartment syndrome (WLCS) and deep venous thrombosis (DVT), which are the most commonly associated adverse events with laparoscopic and robot-assisted rectal surgery.

**Methods:**

A total of 30 healthy participants were included in this study. The pressure (mmHg) applied on various lower limb muscles when using conventional lithotomy stirrups-1 and new type stirrups-2 was recorded in various lithotomy positions; 1) neutral position, 2) Trendelenburg position (15°) with a 0° right inferior tilt, and 3) Trendelenburg position (15°) with a 10° right inferior tilt. Using a special sensor pad named Palm Q®, and the average values were compared between two types of stirrups.

**Results:**

The use of new lithotomy stirrups-2 significantly reduced the pressure applied on the lower limb muscles in various lithotomy positions compared with the use of lithotomy stirrups-1. The most pressured lower limb muscle when using both lithotomy stirrups was the central soleus muscle, which is the most common site for the development of WLCS and DVT. In addition, when using the conventional lithotomy stirrups-1, the pressure was predominantly applied to the proximal soleus muscle; however, when using lithotomy stirrups-2, the pressure was shifted to the more distal soleus muscle.

**Conclusion:**

These results suggest that the new lithotomy stirrups-2 is useful in reducing the pressure load on leg muscles, especially on the proximal to central soleus, and may reduce the incidence of WLCS and DVT after rectal surgery performed in the lithotomy position. Further clinical studies are needed to determine whether the use of lithotomy stirrups-2 prevents these complications in various clinical settings.

## Background

With the evolution of medical technology and improvement in surgical techniques, the number of cases of laparoscopic and robot-assisted surgery for pelvic diseases such as rectal cancer has been increasing [1–3]. However, in cases of narrow pelvis and advanced cancer, the operative time is prolonged, which prolongs the head-down tilt lithotomy position. These factors are believed to cause well-leg compartment syndrome (WLCS), which is a serious complication often requiring emergency surgery with decompression and may result in permanent disability [4–6].

**Fig. 1.**
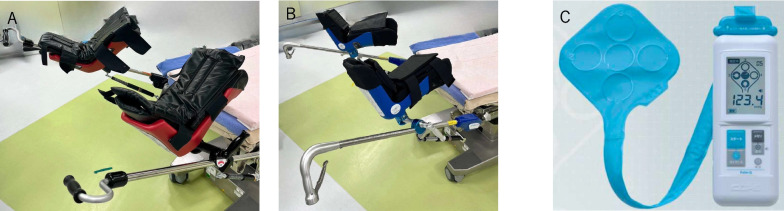
Types of lithotomy stirrups and pressure measuring instruments used in this study. **A** Lithotomy stirrups-1; (**B**) lithotomy stirrups-2; and (**C**) the Palm Q® device for measuring the pressure applied

The development of deep venous thrombosis (DVT) in the lower limbs after the surgical resection of rectal cancer is another important complication [7–9]. The compression of lower limbs by lithotomy stirrups followed by increased intramuscular venous pressure may influence the development of DVT [10, 11]. For two decades, conventional lithotomy stirrups (lithotomy stirrups-1; marketed from December 2000 to March 2022) have been widely used in Japan and other countries [12] as a lower leg fixture in the lithotomy position (Fig. 1A). Thereafter, an improvement in the boot of the stirrups (lithotomy stirrups-2) was approved by the pharmaceutical affairs bodies and has been marketed since October 2021 in Japan.

**Table 1:** Backgrounds of enrolled subjects

item	(*n*=30)	Median (min-max)
Age (years)	35	(21- 56)
Gender (male / female)	19	/ 11
Height (cm)	170.5	(141-186)
Weight (kg)	60	(40.5-98)
BMI (kg/m2)	21.04	(17.1-30.93)
History of venous thrombosis	0	

Lithotomy stirrups-2 (Fig. 1B) is 1.3 times thicker than lithotomy stirrups-1 and is considered to disperse the pressure more evenly, thus preventing load concentration even when body rotation is applied. However, there have been no reports on the detailed examination and measurement of pressure load changes in compartmentalized muscles, such as the central soleus, gastrocnemius, distal soleus, and proximal soleus muscles, with the use of lithotomy stirrups-2.

Therefore, in this study, we measured the pressure applied on lower limbs by two types of leg fixtures (lithotomy stirrups-1 and -2) in 30 healthy participants placed in various lithotomy positions — normal lithotomy position, Trendelenburg position, and Trendelenburg position with a 10° right inferior tilt — using Palm Q®, a portable contact pressure measuring device, attached to the designated areas on the lower limb. The primary aim of this study was to investigate the effect of the use of lithotomy stirrups-2 on pressure dispersal, which may prevent the development of WLCS and DVT, which are serious complications associated with the minimally invasive laparoscopic and robot-assisted rectal surgery.

## Methods

A total of 30 healthy participants were included in this study (Table 1). All participants provided their voluntary written consent to participate in this study after receiving a full explanation. The inclusion criteria were as follows: 1) participants aged 20 years or older at the time of obtaining consent and 2) those classified as class I in the American Society of Anesthesiologists classification of severity of illness.

**Fig. 2:**
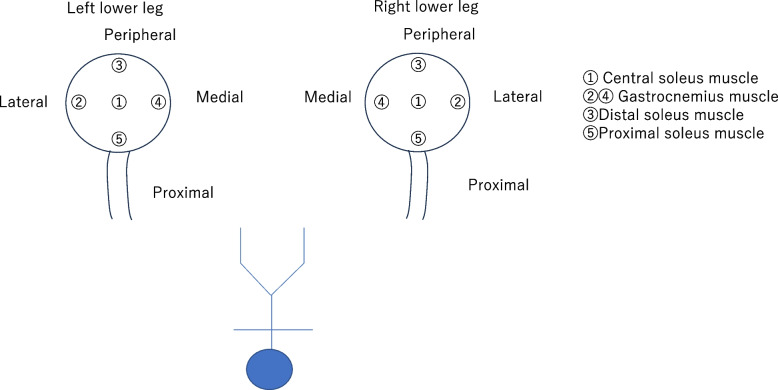
Measurement of pressure applied using the Palm Q® device on the corresponding lower limb muscles. ① Central soleus muscle; ② and ④ gastrocnemius muscle; ③ distal soleus muscle; and ⑤ proximal soleus muscle

The participants were placed in the supine position on the operating table, and both lower extremities were spread and immobilized using lithotomy stirrups-1. The participants were placed in the following lithotomy positions: 1) neutral position, 2) Trendelenburg position (15°) with a 0° right inferior tilt, and 3) Trendelenburg position (15°) with a 10° right inferior tilt. Palm Q ® (Cape Corporation: Yokosuka, Japan), a sensor pad made of urethane with a thickness of 2 mm that can measure the pressure at five points simultaneously, was affixed inside the boots of the lithotomy stirrups fixed to both lower limbs (Fig. 1C). The five sensors of the sensor pad were placed in contact with the central soleus muscle ①, the gastrocnemius muscle ② and ④, the distal soleus muscle ③, and the proximal soleus muscle ⑤ (Fig. 2). After completing the pressure measurement using lithotomy stirrups-1, the procedure was repeated using lithotomy stirrups-2 and pressure measurements were recorded.

The age, sex, height, weight, medical history, and comorbidities of the 30 participants were obtained through interviews as observation items. The pressure (mmHg) was recorded five times on the right and left lower limbs in various positions using the five pressure sensors built into the pad, and the average value was used as the final value. The average pressure values obtained using lithotomy stirrups-1 and -2 were compared. To minimize the affection of the interval between the test, the interval of at least 30 minutes was allowed between the test using the stirrups 1 and 2.

For statistical analysis, continuous variables were compared using the paired t-test (two-tailed test), and nominal variables were compared using the chi-square test. Residual analysis was performed to identify specific points that largely contributed to the chi-square test result. The analyses were performed using IBM SPSS, and a *p*-value of <0.05 was considered statistically significant.

## Results

**Fig. 3.**
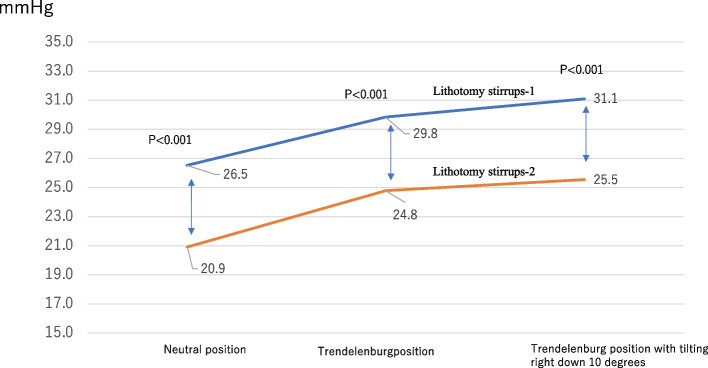
Comparison of average maximum pressures between lithotomy stirrups-1 and -2 use in each lithotomy position

The mean age of the participants was 35 years (range, 31–56 years), and the mean body mass index was 21 kg/m^2^ (range, 17.1–30.9 kg/m^2^). There were 19 males (63.3%) and 11 females (36.6%). No patient had a history of venous thrombosis. The mean maximum pressure values of all participants in each position were compared when using lithotomy stirrups-1 and -2 (Fig. 1). The pressure applied was significantly lower with the use of lithotomy stirrups-2 than with the use of lithotomy stirrups-1 in the neutral position (20.9 vs. 26.5 mmHg; *p*<0.001), in the Trendelenburg position with a 0° right inferior tilt (24.8 vs. 29.8 mmHg; *p*<0.001), and in the Trendelenburg position with a 10° right inferior tilt (25.5 vs. 31.1 mmHg; *p*<0.001) (Fig. 3).

When we evaluated the frequency of the maximum pressure applied according to the type of the muscle, lithotomy stirrups used, and left or right limb, we found that the highest pressure was applied most frequently to ① when using both lithotomy stirrups-1 and -2. The second highest pressure was applied to ⑤ when using lithotomy stirrups-1 and to ③ when using lithotomy stirrups-2 (Tables 1 and 2). A detailed comparison of the average pressures when using lithotomy stirrups-1 and -2 based on the muscle type and lower limb side is presented in Table 3. In the neutral position, the pressure applied was significantly lower with the use of lithotomy stirrups-2 than with the use of lithotomy stirrups-1 on the left lower leg at points ①, ②, ④ and ⑤ (*p*=0.037, 0.045, 0.036, and 0.007, respectively). On the right lower limb, the pressure applied was significantly lower with the use of lithotomy stirrups-2 than with the use of lithotomy stirrups-1 at all points (① [*p*<0.001], ② [*p*<0.001], ③ [*p*=0.048], ④ [*p*<0.001], and ⑤ [*p*<0.001]). When the position was changed to the Trendelenburg position, on the left lower limb, the pressure applied was significantly higher with the use of lithotomy stirrups-2 than with the use of lithotomy stirrups-1 at ③ (*p*=0.005) and ⑤ (*p*=0.013). On the right lower leg, the pressure applied was significantly lower with the use of lithotomy stirrups-2 than with the use of lithotomy stirrups-1 at points ①, ②, ④, and ⑤ (all *p*<0.001).

**Table 2:** Frequency of maximum pressure applied, according to type of muscle, lithotomy stirrups, and left and right side

		Lithotomy stirrups-1	Lithotomy stirrups-2	
Left	① Central soleus muscle	46	49	*P*<0.001
	②④ Gastrocnemius muscle	18	16	
	③Distal soleus muscle	3	21*	
	⑤Proximal soleus muscle	23	4*	
Right	① Central soleus muscle	54	41	*P*<0.001
	②④ Gastrocnemius muscle	23	26	
	③Distal soleus muscle	1	17**	
	⑤Proximal soleus muscle	12	5**	

*Significant difference by residual analysis

**Table 3:** Comparison of average pressure in lithotomy stirrups-1 and 2 (details by muscle, left and right)

Neutral position					Trendelenburg position					Trendelenburg position with tilting right down 10 degrees				
**Left**		**Lithotomy stirrups-1**	**Lithotomy stirrups-2**	***p*** **value**	**Left**		**Lithotomy stirrups-1**	**Lithotomy stirrups-2**	***p*** **value**	**Left**		**Lithotomy stirrups-1**	**Lithotomy stirrups-2**	***p*** **value**
	①	23.8±6.8	20.5±4.9	**0.037**		①	27.0±6.3	25.7±4.3	0.331		①	28.4±7.4	25.0±5.4	**0.044**
	②	21.2±6.5	18.0±5.4	**0.045**		②	25.0±6.4	23.1±4.2	0.168		②	23.5±7.3	20.2±6.1	0.065
	③	17.9±4.5	20..3±5.0	0.059		③	19.5±5.2	23.0±4.1	**0.005**		③	19.5±5.0	22.1±4.5	**0.043**
	④	22.0±5.1	18.6±5.0	**0.036**		④	25.0±6.8	23.2±3.8	0.224		④	27.6±7.3	23.8±4.8	**0.022**
	⑤	20.0±5.7	16.0±5.6	**0.007**		⑤	24.3±6.2	20.5±5.5	**0.013**		⑤	25.6±7.1	20.4±6.2	**0.003**
**Right**		**Lithotomy stirrups-1**	**Lithotomy stirrups-2**	***p*** **value**	**Right**		**Lithotomy stirrups-1**	**Lithotomy stirrups-2**	***p*** **value**	**Right**		**Lithotomy stirrups-1**	**Lithotomy stirrups-2**	***p*** **value**
	①	25.8±6.0	18.1±4.2	**<0.001**		①	28.6±6.3	22.3±4.6	**<0.001**		①	28.3±6.6	23.7±4.3	**0.002**
	②	23.5±6.4	15.8±4.8	**<0.001**		②	26.0±7.1	19.8±4.9	**<0.001**		②	24.4±6.3	19.6±4.7	**0.002**
	③	20.3±5.9	17.4±5.3	**0.048**		③	21.8±5.5	19.9±5.1	0.174		③	21.2±5.8	20.6±4.7	0.068
	④	22.7±4.8	16.2±4.1	**<0.001**		④	25.7±4.6	20.5±4.2	**<0.001**		④	28.8±5.9	23.9±3.8	**<0.001**
	⑤	20.9±6.1	13.6±4.1	**<0.001**		⑤	23.6±5.3	17.7±4.6	**<0.001**		⑤	24.7±4.4	19.3±4.8	**<0.001**

Finally, the pressure was measured in the Trendelenburg position with a 10° right inferior tilt, which is the most frequently used position in rectal cancer surgery.

On the left lower limb, the pressure applied was significantly lower with the use of lithotomy stirrups-2 than with the use of lithotomy stirrups-1 at ① (*p*=0.044), ④ (*p*=0.022), and ⑤ (*p*=0.003) but was significantly higher with the use of lithotomy stirrups-2 at ③ (*p*=0.043).

Similarly, on the right lower limb, the pressure applied was significantly lower with the use of lithotomy stirrups-2 than with the use of lithotomy stirrups-1 at ① (*p*=0.002), ② (*p*=0.002), ④ (*p*<0.001), and ⑤ (*p*<0.001).

## Discussion

The following topics were newly elucidated in the present preliminary research:

1) The use of lithotomy stirrups-2 significantly reduced the pressure applied on the lower limb muscles in various lithotomy positions compared with lithotomy stirrups-1. 2) The most pressured lower limb muscle with the use of both lithotomy stirrups-1 and -2 was the central part of the soleus muscle, which is the most common site for the development of WLCS and DVT, as reported in previous studies [13, 14]. 3) In addition, when using the conventional lithotomy stirrups-1, the pressure was predominantly applied to the proximal soleus muscle; however, when using lithotomy stirrups-2, the pressure was shifted to the more distal soleus muscle.

Compartment syndrome is a condition in which the internal pressure in the fascia-covered muscle compartment (compartment) increases owing to edema or hemorrhage, causing necrosis and fibrosis of the muscle due to microcirculatory or sensory disturbance, nerve paralysis, or renal damage due to elevated myoglobin levels [14].

Delayed diagnosis and treatment can lead to permanent disability, limb amputation, or death. Acute compartment syndrome is defined as intramuscular hemorrhage caused by fracture or trauma or by prolonged compression or ischemia such as cast immobilization [15, 16] and is distinguished from chronic compartment syndrome [17, 18], which is caused by damage resulting from the overuse of muscle groups owing to prolonged exercise. Among the acute compartment syndromes, WLCS is caused by improper intraoperative positioning of the lower extremities and is mostly associated with prolonged surgery in the lithotomy position [19–21].

Halliwill et al. [22] reported that WLCS occurs in 1 out of every 3500 lithotripsy procedures (0.028%); however, in recent years, several studies have reported a higher incidence. In 2009, Tomassetti et al. [23] reported the incidence of WLCS in 373 laparoscopic endometriosis surgeries (0.8%), and, in 2014, Bauer et al. [24] reported that the incidence of WLCS in gynecologic surgery ranged from 0.067–0.28%. Since 2009, we have observed two cases (0.8%) of WLCS in 240 robotic-assisted rectal cancer surgeries at Fujita Health University Hospital. Therefore, to prevent the occurrence of these fatal complications, not only the surgeon but also other paramedical staff must pay close attention to preoperative positioning, intraoperative tilt angles, and monitoring of the lower limb and must perform decompression procedures appropriately. However, the effects of these efforts cannot be quantified, making it difficult to provide clear evidence. In addition, WLCS is highly infrequent, and it is impossible to obtain statistical evidence from observational studies at a single institution. The present study is the first study to scientifically quantify and demonstrate the effect of the use of new lithotomy stirrups-2 on lower limb decompression in lithotomy positions. As shown in Fig. 3, the pressure applied on the lower limb muscle when using lithotomy stirrups-1 increased to 26.5, 29.8, and 31.1 mmHg when the lithotomy position was changed from neutral to Trendelenburg position and to the Trendelenburg position with a 10° right inferior tilt. As most rectal cancer surgeries are usually performed in the Trendelenburg position with a 10° right inferior tilt, we believe that this increase in pressure is one of the causes of WLCS. Furthermore, we quantitatively demonstrated that the use of lithotomy stirrups-2 significantly reduces this pressure.

The present study also revealed that the central soleus muscle is most frequently subjected to the pressure load. Postoperative incidence of WLCS and DVT are most often associated with the soleus muscle, and pressure loading in this area must be considered during rectal surgeries [13, 25, 26]. However, as pressure application on the soleus muscle is not avoidable when using lithotomy stirrups, it is important to improve the quality of the stirrups to reduce the pressure load on this muscle.

We showed that the use of both lithotomy stirrups-1 and -2 resulted in a high frequency of pressure loading on the central soleus muscle, but the actual pressure was significantly reduced with the use of lithotomy stirrups-2 in most lithotomy positions. Furthermore, the use of lithotomy stirrups-2 shifted the pressure load from the soleus muscle to the more distal soleus muscle. DVT is more likely to occur in intramuscular veins that primarily depend on muscle pumping and venous valves than in those that primarily depend on arterial beat. The soleus vein is reported to be more likely to develop thrombosis than the gastrocnemius vein because of the structure of the vein and the muscle. Moreover, soleus vein thrombosis often extends along the drainage veins toward the proximal veins and has been reported to develop lethal pulmonary emboli more frequently than in the gastrocnemius vein [27]. Therefore, prevention of the formation of thrombus in the proximal soleus vein is clinically essential to prevent sudden death due to pulmonary embolism after rectal carcinoma surgery. Based on the results obtained in the present study, we believe that the introduction of the new lithotomy stirrups-2 may reduce WLCS and DVT caused by the lithotomy position.

The present study has some limitations. First, the sample size was small, and the study should be regarded only as an observational study. Second, this article does not prove that new lithotomy stirrups reduce the frequency of WLCS and DVT after surgery, and therefore further large-scale prospective or randomized study is needed to show the true effect of new lithotomy stirrups. Third, the average age of the participants was 35 years old, which is too young age as a candidate for rectal cancer. Fourth, since the diameter of the Palm Q pad is approximately 10 cm and each sensor is equally spaced and the soleus and gastrocnemius muscles have many overlapping areas, it is difficult to completely segregate each muscle of the lower leg to measure pressure load. Forth, However, the pressure load on the lower leg cannot be completely eliminated even with the use of newer stirrups2 as shown in the Table 3. Thus, in the near future, it is desirable to develop a boot pad that is tailor-made to suitably fit the individual patient's body shape (obese or skinny etc.) and its lower legs.

## Conclusion

In conclusion, the new lithotomy stirrups-2 is useful in reducing the pressure load on lower limb muscles, especially on the proximal to central soleus, and may reduce the incidence of WLCS and DVT after the surgery performed in the lithotomy position. A prospective study is currently underway to examine the frequency of postoperative DVT with the use of lithotomy stirrups-1 and -2.

## Data Availability

All the data generated or analyzed during this study are included within the article.

## Abbreviations

WLCS: Well-leg compartment syndrome
DVT: Deep vein thrombosis
